# Ensuring sustainability of polio immunization in health system transition: lessons from the polio eradication initiative in Indonesia

**DOI:** 10.1186/s12889-021-11642-7

**Published:** 2021-09-06

**Authors:** Luthfi Azizatunnisa’, Utsamani Cintyamena, Yodi Mahendradhata, Riris Andono Ahmad

**Affiliations:** 1 Department of Health Behavior, Environment and Social Medicine, Faculty of Medicine, Public Health and Nursing, Universitas Gadjah Mada, Yogyakarta, Indonesia; 2 Center for Tropical Medicine, Faculty of Medicine, Public Health and Nursing, Universitas Gadjah Mada, Yogyakarta, Indonesia; 3 Department of Health Policy and Management, Faculty of Medicine, Public Health and Nursing, Universitas Gadjah Mada, Yogyakarta, Indonesia; 4 Department of Biostatistics, Epidemiology and Population Health, Faculty of Medicine, Public Health and Nursing, Universitas Gadjah Mada, Yogyakarta, Indonesia

**Keywords:** Sustainability, Maintenance, Immunization, Polio, Implementation research, Implementation science, Health system, Decentralization, Health system transition

## Abstract

**Background:**

Sustaining an effective evidence-based health intervention will maximize its impact on public health. Political and governmental reforms impacted on immunization program sustainability both positively and negatively. This study aims to explore the sustainability of polio immunization in a decentralized health system taking lessons learned from a polio eradication initiative in Indonesia.

**Methods:**

We collected qualitative data through in-depth interviews with 27 key informants from various backgrounds at district, provincial, and national levels, consisting of frontline workers, managers, and Non-government Organizations (NGOs). We conducted thematic analysis and triangulated using document reviews. We also conducted member checking and peer debriefing to ensure trustworthiness.

**Results:**

Competing priority was identified as the significant challenge to sustain government commitment for polio immunization and AFP surveillance during the transition toward a decentralized health system. Variation of local government capacities has also affected immunization delivery and commitment at the sub-national level government. The government reform has led to a more democratic society, facilitating vaccine rejection and hesitancy. The multi-sector partnership played a significant role in maintaining polio immunization coverage. Strong and continuous advocacy and campaign were essential to raising awareness of the community and policymakers to keep polio in the agenda and to maintain the high polio immunization coverage.

**Conclusion:**

Competing priority was the major factor affecting high polio immunization coverage during the decentralization transition. Strong advocacy is needed at every level, from district to national level, to keep polio immunization prioritized.

## Background

Sustainability has been defined as “the extent to which an intervention is maintained or institutionalized in a given setting” [1, 2]. Planning for program sustainability is a key contributor to health and development, especially in low-and middle-income countries (LMICs). Maintaining sustainability has been identified as a necessary effort to maximize the public health impacts of an evidence-based intervention [3]. Most concerns about sustainability are related to the premature discontinuation of a program after the initial period of support [4]. Aside from the unmet needs, the discontinuation of beneficial programs is wasteful of human, financial, and technical investment. Moreover, failure to maintain the sustainability of programs in community settings may result in low levels of community support, trust in public health institutions, and support for future programs [3–5]. Throughout all of the elements described in many papers, four general domains of sustainability have been identified: ensuring supportive context (political, organizational, and environmental), capacity building (stakeholders and community), effective partnerships and relationships, and rigorous decision making and planning [6].

Immunization has been long identified as an effective intervention to control vaccine-preventable diseases (VPDs) such as polio. Indonesia introduced polio immunization into the routine immunization program in 1981. Following the World Health Assembly (WHA) 1988, Indonesia initiated a polio eradication initiative called *Erapo (Eradikasi Polio)* in 1991. The Government of the Republic of Indonesia had a strong commitment to implement this policy which resulted in polio elimination in 1995. However, the immunization program faced challenges in maintaining its performance during political and governmental reform in 1998. Global Alliance in Vaccine and Immunization (GAVI), now Gavi the Vaccine Alliance, has defined immunization sustainability as “the ability of a country to mobilize and effectively use domestic and supplementary external resources on a reliable basis to achieve current and future target levels of immunization performance in terms of access, utilization, quality, safety, and equity”. The political and governmental reforms affected the health system and the sustainability of health programs, including immunization [7, 8].

The political and government reform took place in 1998 after decades of authoritarian and centralized government. This reform aimed to establish a democratic government with a decentralized system by mandating provincial and district level government authority. This reform was followed by the reformation of almost all aspects of government, including the health system. Following the government reform, the decentralization system has been enacted since 1999 [9].

Health system reform refers to a process of changing the system of health to improve efficiency. Health system reform had been identified to have an impact on immunization. Reforms likely involved operational changes in immunization management [8]. The decentralized health system provide opportunity to extend the standards developed for immunization to other aspects of primary health care. Thus, it could reinforce good management practices and build up capacity. The health reform also provided an opportunity to consider new funding arrangements for supporting immunization, especially in procuring specialized equipment [8]. Besides the benefits, many obstacles were identified in immunization during the health reform period and transition, including human resource, and organizational, socioeconomic, and legal challenges. Significant changes in the health system and government system reform affected the sustainability of the polio eradication initiative that had been built for years. A previous study suggested that routine immunization suffered a significant fall due to decentralization [10]. Another study demonstrated that decentralization was related to lower levels of childhood immunization coverage [11].

Many studies have reported the impact of decentralization on the health system and healthcare services. However, during the literature review, none has focused on investigating the effects of decentralization on the sustainability of the polio eradication initiative. Therefore, this study aimed to explore the sustainability of polio immunization during health system transition by taking lessons learned from polio eradication initiatives in Indonesia.

## Methods

### Study design

This study is a qualitative case study. In-depth interviews and document reviews were conducted as data collection methods. In-depth interviews were conducted face-to-face and by phone. Phone interviews were conducted with several participants when face-to-face interviews were not feasible due to scheduling and geographical challenges. Reviews of polio related documents and reports were conducted to support qualitative findings and for triangulation. The documents were obtained from online search, libraries, key informants and associated partner institutions such as the Ministry of Health, WHO Indonesia Country Office, Provincial Health Office, and District Health Office.

### Context

Before 1999, Indonesia’s government system was centralized. In 1999, Law Number 22 on Regional Development was implemented, giving full authority to districts/municipalities to rule and be responsible for the governance of their area. During this time, the political and social landscapes were also shifting from authoritarian to democracy and decentralization. Following this political reform, Indonesia’s health system was also transitioned to a decentralized system [9].

The Indonesian health system is supported by public and private providers and financing. The public system is administered through a decentralized government system, with central, provincial and district government involvement. However, in the decentralized health system, the relationship between The Ministry of Health (MoH), Provincial Health Office (PHO) and District Health Office (DHO) is not hierarchical. The district government is not under the provincial government. Instead, each level has its mandates and areas of authority [9, 12].

Since 2001, the number of provinces has expanded from 26 to 34. At the same time, there have been significant regional disparities in health status and the quality, availability, and capacity of health services. This transition affected the capacity of the MoH to implement health programs and maintain integration and alignment across the different levels of the health system. Though decentralized health system has been enacted, the MoH still has a few vertical programs directly delivered at the provincial and district levels, such as immunization [9].

The polio eradication program is under the Directorate General of Disease Control and Prevention (DGDC) and implemented by the Directorate of Surveillance and Health Quarantine (DSHQ) within DGDC. Within DSHQ, polio immunization implementation sits within the Sub-directorate of Immunization, while Acute Flaccid Paralysis (AFP) surveillance is under the Sub-directorate of Surveillance. Meanwhile, the responsibility for vaccine procurement is held by the Directorate of Pharmacy and Health Equipment (DPHE); the responsibility for the health laboratories falls under the Directorate General of Medical Services; and the responsibility for developing information, education and communication (IEC) material is under Directorate of Health Promotion [13].

PHO and DHO have responsibility to supervise hospitals and health centers at the provincial and district level. Local governments are responsible for delivering immunization programs in their areas, while the central government remains responsible for additional immunization activities; providing vaccines, syringes and needles; technical assistance; developing guidelines; monitoring and evaluation; maintaining quality; and training [9, 13].

#### Polio history

**Fig. 1:**
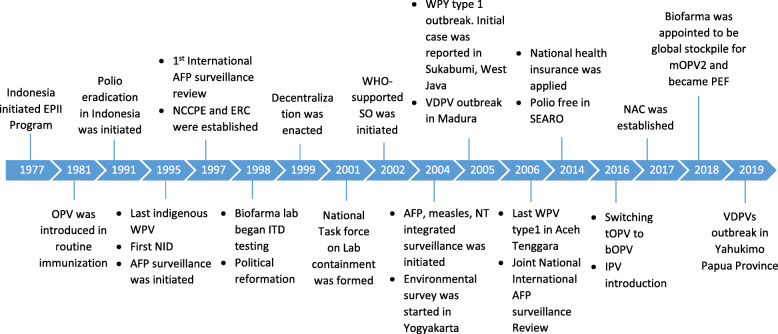
Timeline of Polio History in Indonesia

Indonesia has a long history of polio immunization (Fig. 1). The national immunization program was first introduced in 1973. It was started with BCG immunization and expanded to include TT and DPT in 1974 and 1976. In the following year, 1977, Extended Program in Immunization (EPI) was initiated using WHO global immunization guidelines in 55 health centers. During the same years, EPI Basic Guidelines were developed for Indonesia. In 1981, OPV was added to the EPI with four doses of tOPV. In 1995, the last indigenous poliovirus was reported. After ten years polio free, in 2005, imported WPV was detected in Sukabumi, West Java, and later caused an outbreak with 305 cases reported in several provinces. Apart from the WPV outbreak, there was VDPVs outbreak in Madura, East Java. The outbreak was successfully contained. The last poliovirus was detected in Aceh Tenggara in 2006. Indonesia, along with SEAR countries, was granted polio-free certification in 2014. In 2016, the government implemented a switch from tOPV to bOPV and introducing IPV [14].

### Study participants

Purposive snowball sampling techniques were applied to recruit study participants with rich information on polio eradication in Indonesia. Study participants were from national and sub-national levels and frontline workers from various affiliations and backgrounds. We selected the participants who had worked on polio eradication for at least 12 months between 1988 and 2018. This study is under the Synthesis and Translation of Research and Innovation from the Polio Eradication (STRIPE) Project. Initially, the STRIPE project has engaged the Ministry of Health at the beginning of the project. Before conducting in-depth interview, we had conducted the survey in the six selected provinces which were suggested by the MoH. From the survey, we had identified the key persons in the polio eradication program in Indonesia at national and sub-national levels. From these key persons, the snowball sampling was rolled. The final number of participants was achieved after reaching data saturation when we no longer found new information regarding the topic.

### Study setting

The study was conducted by recruiting participants at the national and sub-national levels. Six provinces were selected purposively to represent major challenges faced by polio eradication in Indonesia. They are Yogyakarta, West Java, Banten, East Java, Aceh and East Nusa Tenggara. Yogyakarta was chosen because it was the province where IPV was piloted and the first province to switch from OPV to IPV. West Java and East Java were selected due to the WPV and VDPV outbreaks that occurred in 2005. Banten was chosen because the WPV outbreak spread to Banten, and according to MoH reports, immunization rejection was highly prevalent. Aceh was selected because the last WPV case was found in Aceh province in 2006. Finally, East Nusa Tenggara was selected as representative of the eastern part of Indonesia, with major geographical and infrastructural challenges. In-depth interviews were conducted between January–March 2019. In addition, document reviews were conducted from December 2018 to May 2019.

### Data collection procedures

Potential participants (polio key informants) were listed. Interviews were scheduled by making appointments with them. The study was explained to participants, and they were given time to discuss and clarify. If participants agreed to be interviewed, they were asked to sign informed consent. For phone interviews, verbal consent was sought. Interviews were conducted using an interview guideline piloted to the immunization and surveillance program managers at PHO of Yogyakarta before data collection. The interviews guideline has been published elsewhere as a supplemental information [15].

 Interviews were performed in Bahasa Indonesia by two study team members who have a qualification of master in public health and vast experience in the qualitative study and have been trained on Good Health Research Practices [16]. Interviews were recorded using a digital audio recorder. Recordings were transcribed into verbatim transcripts by professional transcribers. Quality checks of the transcript were performed by comparing the transcript, the recordings and the field notes conducted by the study team. Finally, thematic analysis was applied to analyze the data.

Confidentiality of the data was ensured by making the transcript and data in the final report anonymous. The ethical clearance was obtained from the Medical and Health Research Ethics Committee (MHREC), Faculty of Medicine, Public Health and Nursing, Universitas Gadjah Mada, Indonesia (Approval Number: KE/FK/0757/EC/2018) and John Hopkins Bloomberg School of Public Health (JHSP) Institutional Review Board (IRB) (IRB number: IRB00008721)**.**

### Analysis

Thematic analysis was applied, and transcripts were coded into meaningful units. Similar codes were grouped into categories, and themes were generated from these categories. Linkages between themes were then identified. A sustainability framework by Schell (2013) was applied for analysis [17]. The analysis was performed using OpenCode 4.03 software (https://www.umu.se/en/department-of-epidemiology-and-global-health/research/open-code2/). Peer debriefing, member checking and triangulation were conducted to ensure trustworthiness of the data.

## Result

### Participants

We listed ten potential participants for in-depth interviews. During the data collection process, the participants recommended other potential participants and snowball sampling was applied. In total, we interviewed 27 key informants consisting of 16 from the national level, seven from the sub-national level, and four frontline workers from various institutional backgrounds (Table 1). Face-to-face interviews were conducted with 22 participants, and phone interviews were conducted with 5 participants.

**Table 1:** Characteristic of Key Informant Interview (KII) Participants

Variables	Group	N
Age	30–39	1
	40–49	6
	50–59	10
	≥60	10
Sex	Male	12
	Female	15
Polio related affiliation	Central level government	11
	Provincial government	4
	District government	2
	Frontline worker	4
	NGO	1
	Partners (WHO, UNICEF)	5
Interview method	Face-to-face	22
	By phone	5

### Sustainability components

**Fig. 2:**
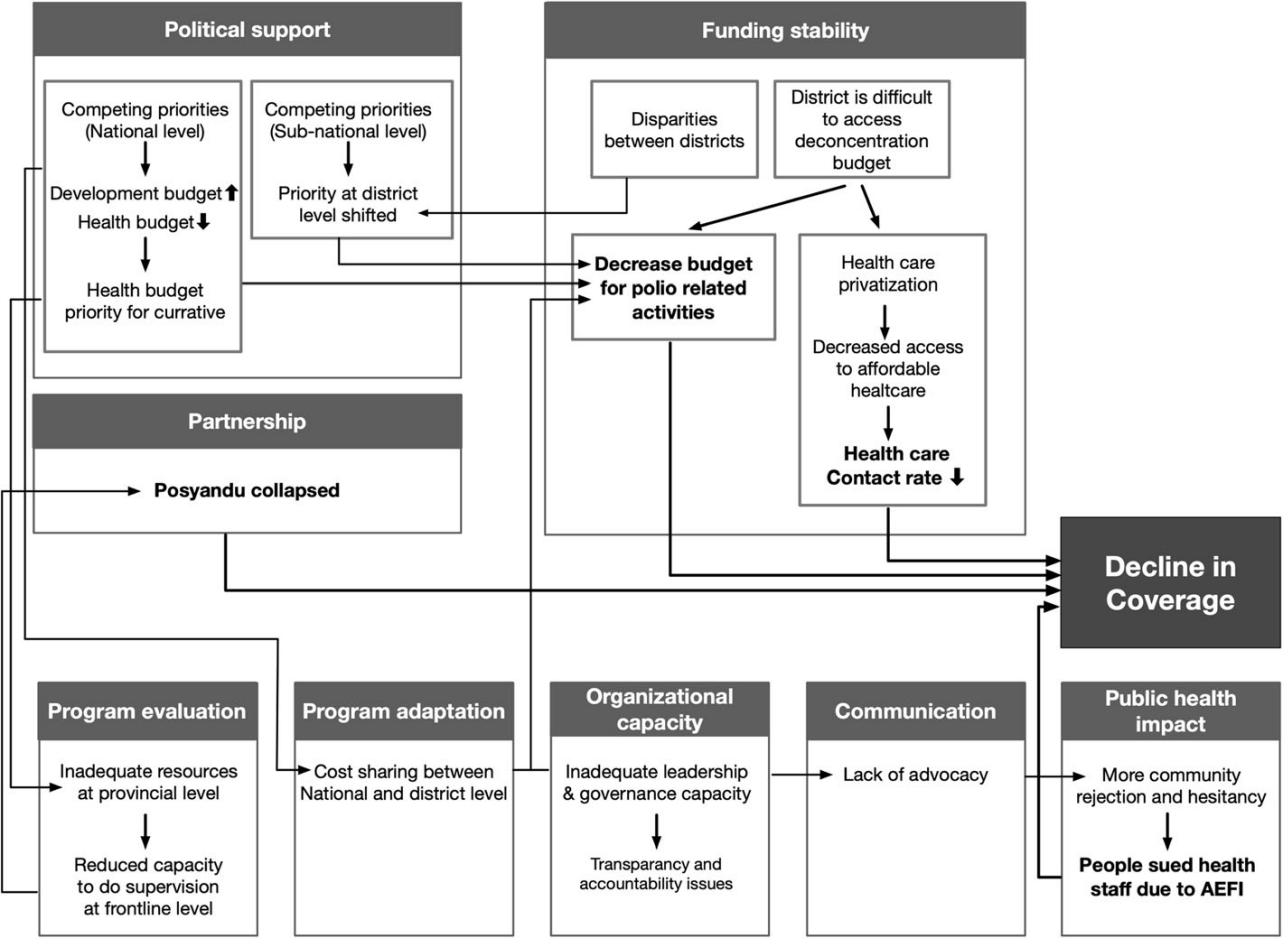
Problem Identified in Polio Immunization Sustainability

This study found that sustainability components affected each other and led to declining immunization coverage during health system transition, summarized in Fig. 2. Using a predetermined sustainability framework, this study developed nine themes and 18 categories (Table 2).

**Table 2:** Themes and Categories Developed from the Study

No	Themes	Categories
1	Political support	Competing priority at the national and sub-national level
		Disparities at the subnational level
		The preventive measure was not a priority in health budget allocation
2	Funding stability	Decreased health budget during the transition
		Low health budget allocation at the sub-national level
3	Partnership	Collapsed Puskesmas (Primary Health Center - PHC) and Posyandu (Integrated health post)
4	Organizational capacity	Insufficient leadership capacity among policymakers at the sub-national level
		Differences in human resource capacity at the national level before and after decentralization
5	Program adaptation	Adaptation of funding sources
6	Program evaluation	Supervision of district was decreased
7	Communication	There was a gap in advocacy capacity among the sub-national levels
		Decreased socialization
8	Public impact	Vaccine hesitancy
		Legal issue due to AEFI
		Involving key persons as a strategy tackling anti-vaccine movement
		NID was perceived as the biggest community movement in health
9	Strategic planning	No significant impact of decentralization on polio strategic planning
		The characteristics of the polio eradication initiative as a contributing factor for the success of the program

#### Political support

##### Competing priority at the national and sub-national level

**Fig. 3:**
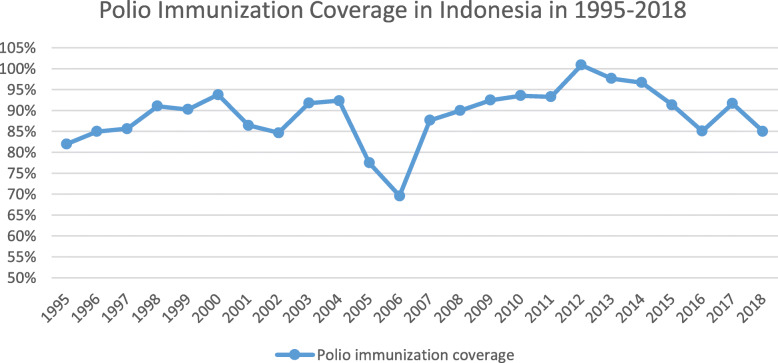
Polio Immunization coverage from 1995 to 2016. 1995, 1996, 1997, 1998: 27 provinces (East Timor still included). 1999: 27 provinces (East Timor was independent, North Maluku was established). 2000: 30 provinces (Banten, Bangka Belitung, and Gorontalo were established). 2001: 31 provinces (West Papua was established). 2002: 32 provinces (Riau Islands was established). 2004: 33 provinces (West Sulawesi was established). 2012: 34 provinces (North Kalimantan was established). *Sources: Sub Directorate of Immunization, MoH of Indonesia (unpublished)*

**Fig. 4:**
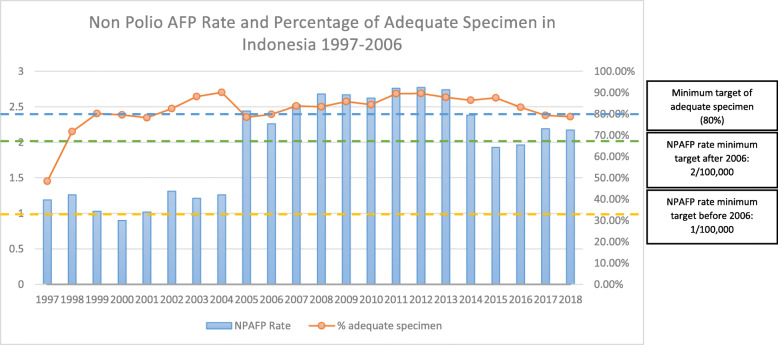
Non-Polio AFP (NPAFP) Rate and Adequate Specimen Percentage 1997–2018. *Source: MoH of RI Decree Number 483/MENKES/SK/2007 on AFP Surveillance Guideline; Indonesia Health Profile 2007–2018*

Competing priority has been identified as the major challenge to sustain polio-related activities during a health system transition. Priority in districts changed; therefore, not all districts allocated adequate amounts of money for polio eradication, resulting in decreased quality and quantity of polio-related activities. Moreover, during government transition, most of the national budget was allocated for political and governmental purposes. Therefore, the health budget was cut (Fig. 2). This impacted on polio immunization coverage in the following years. It was reported that the coverage dropped, and then the outbreak occurred in 2005 (Fig. 3). The Non-Polio Acute Flaccid Paralysis (NPAFP) rate was also affected with a slight decrease during the transition (Fig. 4). However, it increased dramatically in 2005 due to the WHO supported Surveillance Officers (SOs) in every province, initiated in 2002. The NPAFP is an indicator for the sensitivity of surveillance acute flaccid paralysis (AFP), a polio symptom. The cut off is one case of AFP per 100,000 (1/100,000) children under 15 years old. If the NPAFP rate is less than 1/100,000, it indicates a missing AFP case in the population [18].

During the transition from a centralized to a decentralized system, maternal and child immunization coverage and availability remained pressing issues at the district level in Indonesia. Complete child immunization in most districts in Indonesia was below the WHO recommendation threshold of 80% [12]. In 2002, complete child immunization coverage in central java (Cilacap, Rembang, Jepara, Pemalang, Brebes) and East Java (Trenggalek, Jombang, Ngawi, Sampang, Pamekasan) was below 51%, with the lowest level at 9% in Sampang district, East Java [12].

*“…with democracy process in the district, they choose their leader (mayor), of course, the priority of each district is different… The most important thing is to convince the policymakers both at central and district level that this (polio) is a priority, this is an investment and will give huge impact” (Informant 9, Manager at National level).*

##### Disparities at the sub-national level

The reforms also caused disparities between local governments. Disparities become a threat to health due to the new leader's lack of understanding and awareness around funding for health services; this is especially true in poor districts or municipalities. To ensure that local government undertook certain public measures, the MoH issued a decree in 2005 pertaining to 26 types of minimum/essential public health services that the local government must perform. Of these 26 services, 16 were related to public health, such as maternal and child health, promotion and prevention of prevalent diseases, school health and disease surveillance [19]. However, five years after enacting this policy, it was reported that not all district governments applied all of the indicators mentioned in this policy [19].

##### The preventive measure was not a priority in health budget allocation

Many local governments were more interested in strengthening curative health care, such as constructing new facilities or refurbishing existing hospitals, rather than strengthening the primary care infrastructure. In addition, local governments are more concerned about the shortages of medical officers rather than closing the gaps for the deployment of public health professionals at the grassroot level [19].

*“…we conduct advocacy to the government to develop minimum service standard (SPM) where the districts have to have indicators for polio. Though it is applied for immunization, I think it is not powerful enough” (Informant 17, Technical Assistant at National Level)*

#### Funding stability

##### Decreased health budget during the transition

At the central level during the transition, the national budget was concentrated for government reform. The health budget was reduced, and the priority was curative. Therefore, the budget for promotion and prevention was decreased (Fig. 2). This severely impacted on immunization supervision and surveillance. The supervision for immunization received less attention and less funding. Therefore, the coverage of polio immunization decreased during the transition (Fig. 3). Moreover, the polio-free status obtained since 1995 made the government complacent about the AFP surveillance; thus, the NPAFP rate decreased during the transition (Fig. 4).

*“…during the transition, government focus was on funding the government reform. Health budget was cut. Moreover, the priority was for curative…” (Informant 25, Manager at National level)*

Even though a decentralized health system has been applied, vaccine procurement has remained centralized. Funds for procuring and supplying regular vaccines for immunization programs were mainly sourced from the APBN (National Budget and Expenditure) and managed by the Director-General of Pharmaceuticals and Medical Devices, MoH. For the delivery, MoH shared the costs with the district governments.

*“…the difference is that now, programs become more integrated and cost-sharing is applied” (Informant 5, Technical Assistant at National level)*

##### Low health budget at the sub-national level

Decentralization allowed the local government to develop and finance local initiative health programs. However, implementing health programs at local level depends on local fiscal capacity, regulation, and political process. Meanwhile, one of the impacts of decentralization led to widening the fiscal capacity gap between local governments. However, in both poor and rich local governments, the health budget from the general allocation fund (Dana Alokasi Umum/DAU) and APBD was not enough to fund the healthcare services. Moreover, budget constraints were more common in several districts after decentralization due to public health budget reduction [12].

**Fig. 5:**
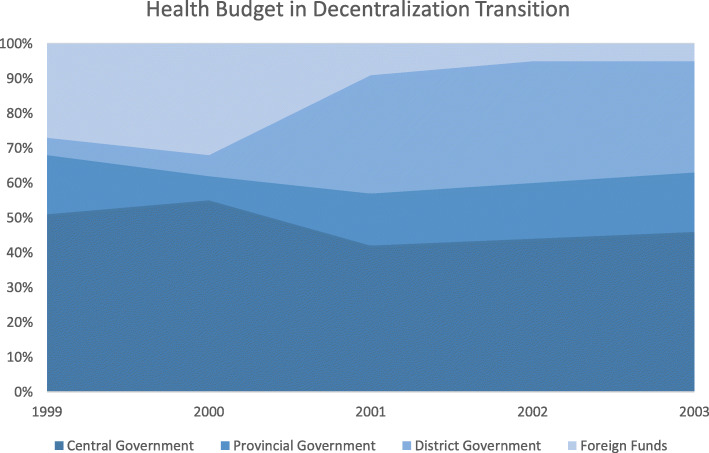
Health Budget Sources in Decentralization Transition. *Source: Desentralisasi Kesehatan (2008)* [20]

The inadequate budget for health at local government resulted in disruptions of program implementation at the local level and thus resulted in lost coordination within the health system. As a result, the central government initiated to provide de-concentration budget through the Specific Allocation Fund (DAK - Dana Alokasi Khusus) for the health sector. Therefore, the central government still funded the highest health budget during the transition (Fig. 5).

The central government established minimum service standards to standardize which public services must exist, including immunization. Immunization is also included in Children Protection Laws, Health Laws, and Regional Government Laws stating that immunization is a must for all children in Indonesia. These regulations required every district government to allocate a budget for immunization. Unfortunately, around half of the districts in Indonesia did not comply with the mandated 10% of the local budget for health. This affects the allocation for immunization service delivery, increasing immunization outreach, and maintaining cold chain equipment [7].

#### Partnerships

##### Collapsed Puskesmas (primary health center - PHC) and Posyandu (integrated health post)

**Fig. 6:**
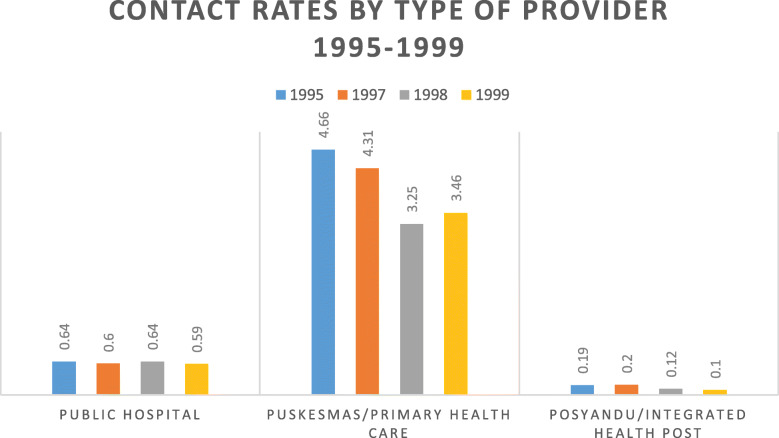
Contact Rates by Type of Provider 1995–1999. *Source: Lieberman, 2002 *[22]

Community mobilization is pivotal in immunization programs. Front line workers at PHC and integrated health posts at the village level, where immunization was delivered, played an essential role in community mobilization. However, the lack of sufficient health funding at the district level has encouraged more Puskesmas to become self-funded by instituting additional charges for service delivery. As immunization was primarily delivered at PHC, the lower-income families could not afford the additional health service fees and withdrew from this facility, further jeopardizing their health status. Moreover, during the decentralization transition, many people financially suffered from the monetary crisis [21, 22]. Figure 6 demonstrates that the contact rates to the public hospital, PHC and Posyandu decreased during the transition. The contact rate is a proportion of new visits in a population per year. It is a performance indicator of healthcare facilities [22].

Furthermore, in 1997, Posyandu attendance by children under 5 was 57%. It decreased further in 1998 to just 42%. Similar trend was also reported in Susenas (National Socio-Economic Household Survey) 1995, 1997 and 1998. The province-specific surveys during the crisis revealed low contact rates for public facilities and a large number of Posyandu inactive [22]. This affected immunization coverage. In the following years, the coverage dropped, and the outbreak occurred in 2005 (Fig. 3). This shows how vital community engagement was for immunization.

*“…In 98, we experienced a multi-dimensional crisis, monetary crisis. Therefore, Mr President had to step down. Back then, our strength for immunization was Posyandu (integrated health post), after the crisis, Posyandu collapsed” (Informant 4, Former Manager at National Level)*

#### Organizational capacity

##### Insufficient leadership capacity among policymakers at the sub-national level

The local capacity and political process influenced the development of local health programs. During the transition, it was reported that the capacity of local government in planning, budgeting, and utilizing their budget effectively and efficiently were not adequate. The local government’s actions in allocating insufficient funding to health budgets for health might be due to poor judgment in decision-making. This disparity hindered local progress in developing capacity [23]. In addition, inadequate leadership and vision among bureaucrats at the local level were identified as major factors that facilitated the local government to continue implementing the old system even after decentralization, rather than answering the current health-related needs and problems, such as immunization and polio.

*“Decentralization is necessary, but it was supposed to be well prepared. Capacity building for the policymakers at the district level need to be conducted before the enactment. From my point of view, districts capacities were not ready for decentralization. They were still dependent to the central level” (Informant 6, Manager at National level)*

##### Differences in human resource capacity at the national level before and after decentralization

Several informants reported differences in terms of human resource capacity at the national level before and after the government reform. In addition, prominent leadership and governance issues were identified during the decentralization transition; including issues around transparency, accountability, health strategy, guideline implementation, and system design [12], which affected the health-related decisions and policies they made.

*“…The quality of human resources decreased, and that nepotism emerged. Smart people, and should be promoted, were pushed away. Those who could not stand anymore resigned and moved to WHO, UNICEF. I was really sad. Those who got promoted are those who did not have any achievement” (Informant 6, Manager at National level)*

#### Program adaptation

##### Adaptation of funding sources

During the transition, the most significant change was the funding source, as previously it had been the central government that funded the program. After decentralization, the program fund was the responsibility of the national and sub-national governments together. However, there was still a division of responsibility. National Government was responsible for procurement and providing guidelines; the provincial government was responsible for the supervision and technical assistance; and, district government was responsible for operations and delivery. Each responsibility was funded by each level budget. However, this funding stream may not be smooth and adequately allocated. Budget allocation has been explained in the funding stability section.

#### Program evaluation

##### Supervision of district was decreased

In the decentralized health system, supervision from the central level was shifted to the provincial level. Therefore, supervision of the districts was conducted by province government. However, as all provinces do not have adequate resources, supervision of the districts or municipalities became a challenge. Therefore, supervision of the districts, especially for surveillance, decreased within the decentralized health system (Fig. 2).

*“What’s the impact of decentralization? Economic became number one, efficiency. Budget was cut. Health budget was cut. Therefore, the quantity of supervision to the health post decreased” (Informant 25, Technical Assistant at National level)*

*“With the decentralization enacted, the central level cannot directly supervise district; the supervision is only up to province level. The province is the one that has the responsibility to supervise the district. This responsibility is also a challenge because provinces do not have sufficient resources to do so. Therefore, the supervision on AFP surveillance performance decreased” (Informant 3, Technical Assistant at National level)*

Coordination and review meetings were held regularly at the provincial and national levels to maintain communication of polio networks and evaluate AFP surveillance performance. This activity was supported by external funding (WHO) by hiring a surveillance officer (SO) at the provincial level, which started in 2002. The term of reference of SO was terminated in 2014.

#### Communications

##### There was a gap in advocacy capacity among the sub-national levels

As there was competing priority at the district level after decentralization, continuous and robust advocacy for polio immunization became essential. Advocacy should be conducted at the national level (within the MOH and other Ministries such as Home Affairs and Planning) and the provincial and district/municipalities level. Unfortunately, the capacity for advocacy within the sub-national governments varied. This considerable gap requires capacity building for advocacy. An advocacy consultant was also hired to plan effective advocacy strategies.

*“What we can do is convince the policymakers… thus, advocacy has to be our mandatory activity. However, the capacity to conduct advocacy seems to be insufficient. We need a motivator, communication specialist, advocate to convince the local government, local representative board, to allocate resources for polio” (Informant 3, Technical Assistant at National level)*

#### Public health impacts

##### Vaccine hesitancy

The first NIDs conducted in 1995 were very festive and engaging as most people voluntarily participated in this event, although some hesitancies existed in a small percentage of people. However, after decentralization, where freedom of speech was assured and information was more freely spread, there was more rejection to immunization. For example, during a mop-up campaign in 2005, media incorrectly blamed the polio vaccine for several coincidental adverse events during the first round of immunization, causing misunderstanding and suspicion among the public.

##### Legal issue due to AEFI

All vaccines which are used in national immunization program are safe and effective if it is used appropriately. However, they still possess the risk to have an adverse event after vaccination. This adverse event is called Adverse Event Following Immunization (AEFI). It may range from mild to severe. This adverse event may cause public questioning on vaccine safety. Therefore, the investigation to determine AEFI is essential, and it is built in an AEFI surveillance system. The aim of AEFI Surveillance is to detect, correct and prevent immunization program fault, identify a potential problem in certain vaccines, prevent false accusations in coincident events, maintain public trust in the immunization program, identify likely unintended events and develop hypotheses that will be tested with study, estimate the prevalence of AEFI in certain population, and contribute in developing and adjusting contraindication, risk/benefit analysis and information for health workers who deliver immunization and patients [24].

After decentralization, democracy was more widely implemented, and the awareness of freedom of speech increased. This gave rise to legal issues known as an AEFI. After decentralization, the number of health staff who were sued due to AEFI increased. This made health staff afraid to deliver immunization services, and they requested protection to carry out their duties. The strategy taken by the MoH was to develop national immunization guidelines as Ministry Decree. Previously, the guidelines were signed by the authority at the directorate-general level, which was not strong enough to become a legal basis for health staff carrying out their responsibilities. This change enabled health staff to have legal assurance when they work in adherence to the guidelines.

*“…in a centralized era, we did not care about the legal standing of regulation, so we only made national guidelines signed only by the director-general. When AEFI occurred, there was no fuss and suing or legal action. After the reformation, due to arisen legal issues, health staff became afraid to give the vaccination. They pushed us to develop national guidelines as MoH decree” (Informant 6, Manager at National level)*

##### Involving key persons as a strategy tackling anti-vaccine movement

Many strategies and measures were implemented to tackle the negativity against vaccinations, such as using the role of professional organizations to take action against doctors who opposed vaccines and using multi-modal interventions to raise the awareness of the community.

*“I even attended the seminar on anti-vaccine to counter their arguments. I challenge that person to argue with scientific evidence. I don’t know how I could be fearless back then, hahaha” (Informant 5, Manager at National level)*

Sensitization of community and stakeholders was intensively conducted during the polio campaign. Ulama, public figures, community leaders and other champions were involved in socialization to convince the community that immunization is very important. Various media sources were used for community sensitization, such as roadshows, printed media, mass media, electronic media, and social media, to counter the negative campaign against immunization that intensified after decentralization. However, most of the informants stated that the quantity and integration of sensitization efforts have decreased.

*“…in socialization, we engaged MUI (Indonesian Ulama Council) to give endorsement (fatwa)… we also made polio campaign in TV starred by celebrity...We engage many brands to support the campaign by providing merchandise. We engage the Ministry of Information and Communication to make regulations for those who want to advertise on TV must convey a little polio message. I think that worked” (Informant 5, Manager at National level)*

##### NID was perceived as the most prominent community movement in health

Though routine immunization has been provided since 1977, the peak of the immunization campaign came with the first NIDs in 1995. This is because it was so exciting and has been claimed as the largest community mobilization for health in Indonesia. In addition, the eventful NIDs provided a strong impression upon the community, and the polio eradication campaign increased community awareness on overall immunization, extending further than just polio. Therefore, the community perceived that immunization is a health need, not simply an enforced obligation.

*“Massive polio campaign has increased the awareness of the community on immunization. Immunization has become their needs” (Informant 1, frontline worker)*

#### Strategic planning

##### No significant impact of decentralization on polio strategic planning

There was no significant impact of decentralization on polio strategic planning. Most of the informants mentioned no difference in the polio program before and after decentralization because the Indonesian government followed a global polio policy. The implementation of polio immunization followed the updated recommendation from WHO. In 2012, the World Health Assembly declared polio as a public health emergency and expressed the need for a comprehensive endgame strategy. As a result, WHO developed a polio eradication and endgame strategic plan for 2013–2018. Indonesia started to implement the polio endgame strategy to maintain polio-free status and achieve the global eradication target in 2020. Following the updated strategic plan, Indonesia switched the polio vaccine from tOPV to bOPV and introduced one dose IPV to enhance and strengthen the immunization program. Poliovirus containment and environmental survey were also added into the activities.

*“We have prepared for the transition process. The process has been run well enough. The document has also been developed. Our roadmap has also been adjusted to the global roadmap on polio eradication. We have implemented switching, improving surveillance, and improving laboratories’ capacity to ensure the eradication process succeed” (Informant 9, Manager at National level)*

##### The characteristics of the polio eradication initiative as a contributing factor for the success of the program

Regardless of many challenges faced during the implementation, most informants mention that the success of the polio eradication initiative was because of the characteristics of the program. Clear and detailed plans, targets, strategies and impacts were identified as the factors that facilitated the implementation of the polio eradication initiative. This clear detail also attracted multi sectors and partners to become involved in polio-related activities.

*“Polio eradication has a clear, detailed program, clear goal and target. With the same goal, the role of each actor was also clear, so that it attracted partners to involve…” (Informant 21, Polio Partners at National level)*

## Discussion

As part of the STRIPE project, this paper focuses on how decentralization affected the polio eradication initiative. Previous findings shared about the escalation process of polio immunization in Indonesia, yet, in this paper, there is an added value of more comprehensive activity in polio eradication; the AFP surveillance system [14].

The sustainability of the immunization program may have been compromised due to political, economic, and structural transitions during the reform era. Competing priority has been identified as a major factor leading to several challenges in immunization sustainability, such as decreased budget, supervision, priority on preventive efforts, also inadequate leadership capacity. In addition, vaccine hesitancy was also reported more after reformation due to the implementation of democracy. As polio has been included in routine immunization since 1981, it was also affected by the transition.

Competing interest for public funds as well as economic slowdowns during reformation affected immunization financing and sustainability. Previous studies suggest a clear association between decentralization and a weakened immunization program [7, 25]. A previous related study shows that more than half (N: 323) actors were involved in the polio eradication program mentioned that external factors such as politics, social and technology were the most challenging during the transition [14]. Increased autonomy at the sub-national level opens doors for an increased number of actors to affect the prioritization of immunization programs while also introducing new challenges for ensuring high performance on national immunization. Sub-national units’ political will and leadership may be the only and most important factors related to immunization performance [7]. Without proper accountability, a decentralized health system can negatively affect immunization and other health programs. For example, Indonesia and The Philippines, considered the most heavily decentralized countries in the Asia Pacific, had poor program performance, which led to decreasing immunization coverage rates [7].

Immunization is still vertically funded by the central government, as shown in the prioritization of immunization in the country. However, sustaining the immunization program requires more than relying on macroeconomic growth and political prioritization [7]. There is a range of potential approaches to improve prioritization in a decentralized health system. First, we can engage with sub-national governments as advocacy partners regarding immunization financing or second, to support research to analyze how current center-local relationships exist. Third, we can direct engagement for improved outcomes by promoting ongoing policy and practice dialogue at both national and national levels sub-national level [7]. However, the study results suggest that the advocacy capacity of local government was lacking during the transition and continued to do so until recently.

The partial division on vaccine procurement and service delivery responsibility between national and sub-national levels has led to uncertain program ownership (possibly exacerbated by differing priorities at the local level) and has almost certainly played a part in the stagnation of immunization coverage since decentralization [26]. Previous studies have identified that the weaknesses of immunization during the decentralization transition were due to challenges in budgeting for vaccine purchase, national procurement practices, the performance of national regulatory agencies, and technical capacity for vaccine planning and advocacy [7, 27]. This result is supported by our previous paper that immunization scaling up was hampered due to health system and government transition [14].

Establishing immunization policy and legislation for a standard of service delivery is also essential to build a strong immunization program [28]. Moreover, more private healthcare facilities were established during the transition; thus, private providers developed higher prominence than those from the public sector. Therefore, the guideline for immunization delivery is very important as a standard and to ensure the quality of delivery among various providers [7]. Several previous studies reported that SPM (minimal service standard) in the health sector could improve health status and improve family welfare. However, implementation for immunization and family planning is still lacking and requires improvement to increase performance [29, 30].

In terms of the sustainability of financing for health programs, the country is pushing to diversify their resources. Diversified sources of funding help to mitigate the risk of political maneuvering or economic downturns, as well as open avenues to increase the overall health budget. As most of the expenditure of immunization programs are on the procurement of vaccines, operational needs can often have less priority. The responsibility of setting priorities is shifting to domestic stakeholders, which may assist this. However, the capacity of sub-national governments in achieving targeted coverage also varies. Although not all practices and approaches implemented in the high coverage area are replicable in other regions, basic planning, budgeting, and delivery functions should be improved in low-performing districts and provinces. Ongoing coaching by high-performing areas to copy their approaches and apply improved accountability mechanisms should be in place [7].

As decentralization aims to manage resources more efficiently based on each district’s needs, the integration of health programs becomes more prevalent for efficiency. Integration of immunization programs with other public health services, such as breastfeeding, maternal nutrition, community midwifery etc., are more effective. A commonly integrated program allows health workers of various units to take up immunization related activities as their responsibility [28].

Many studies have identified community ownership and mobilization as essential for intervention sustainability, at both the beginning of and during the intervention implementation. Involving stakeholders and providing them with a sense of ownership in intervention proved beneficial for a variety of reasons [31]. A previous study suggests that community-provider partnership has been proven to increase immunization coverage [32]. Another study also indicates that increasing the number of *Posyandu* (integrated health post) per 1000 population significantly improves the probability of children receiving full immunization, while increasing the number of hospitals and *Puskesmas* (Primary Health Care) has no significant effect [26]. Posyandu (integrated health post) is a community-based health post managed and delivered by mothers and organized at the sub-village level. Posyandu is essential in monitoring mother and child health. Posyandu is supported by village a midwife who serves as health personnel [33]. Puskesmas (Primary Health Care) is a primary healthcare facility at a sub-district level that organized individual healthcare services and public health services by emphasizing promotive and preventive measures to improve community health status [34]. Before decentralization, Posyandu and Puskesmas played an essential role in achieving Universal Coverage Immunization (UCI) [14]. However, during the transition, the health system had to adjust to the new system. The preventive and promotive measures delivered by Puskesmas and Posyandu were neglected therefore result in decreasing immunization coverage.

Volunteerism is also essential in intervention sustainability. Community volunteers perceived their role in the program similar to other community health workers. Thus, they showed good ownership of the intervention and were ready to take on various responsibilities to continue the intervention [28, 31]. Non-governmental sectors also play a significant role in immunization services, regardless of whether the government sector proactively engages them [31].

High-quality data are pivotal to empower immunization program management, increase vaccine uptake, and reach unvaccinated children. Meanwhile, ensuring the quality and effectiveness of surveillance and public health response is a critical challenge in developing countries in an environment of decentralization. Several health systems barriers constrain the intervention’s effectiveness in influencing data availability, analysis, and response [35]. Utilization of traditional methods, such as home-based vaccination recording, and newer technology, such as information communication and technology tools, are reported to improve data quality on timeliness and accuracy while also contributing to improved immunization coverage [28, 36].

Delegating responsibility to various community workers is vital for the sustainability of an intervention. In addition, better monitoring and program evaluation is needed to sustain interventions [31]. Besides regular coordination meetings at the national, provincial, district and health facility level, involving the private sector, organizations, community leaders, and volunteers can provide timely feedback to improve the immunization delivery services [28].

The lack of community awareness or education regarding the health issues also impeded successful implementation and intervention sustainability. Vaccine hesitancy has been reported in more than 90% of countries in the world. In contrast, media platforms (including social media) have been identified as enormously influential in the spread of vaccine hesitancy. Pediatricians and family doctors have a crucial role in convincing parents of the benefits of vaccination. However, pediatricians and family doctors acting alone are insufficient to overcome vaccine hesitancy. Governments and health policymakers also play an essential role in promoting vaccination, educating the general community, and implementing policies that reduce the public health risks associated with vaccine hesitancy [37, 38].

Community mobilization has been central to NIDs by mobilizing various community organizations; however, this social mobilization was not utilized to improve the chronic problems with routine immunization and surveillance [38]. To address these problems, having champions was critical to ensure sustainability. The organizational representatives that reported high champion effectiveness were most likely from organizations that sustained health interventions [39]. Our previous publication mentioned that community health education and mobilization supported scaling up achieving the immunization targeted coverage during National Immunization Days (NIDs) [14].

Information, Education and Communication (IEC) strategies have been widely used and showed positive results. Various media on polio campaigns have been identified to have a significant impact, such as radio, television, religious organizations, and interpersonal communication between caretakers and community leaders and health workers [40]. The lesson learned from the communication strategy is the need for integrated media, especially when communities are filled with negative rumors or reject vaccination [40]. Utilizing the integrated and combination of all types of communication channels provides a better chance to change mindsets than using a single channel approach [36]. Communication programs for polio eradication have made a number of contributions for capacity building, such as developing micro plans, organizing social mobilization, conducting advocacy among policymakers, dealing successfully with negative campaigns and resistance, and identifying hard-to-reach populations [40].

The degree to which vaccinations provide broad public health benefits is stronger than for other preventive and curative interventions [41]. Government leadership, evidence-based programing, country-driven, comprehensive, and annual operational plans, community partnership, and strong accountability systems are critical for programs. Polio eradication has illustrated that these can be leveraged to increase immunization coverage and equity and enhance global health security [41, 42].

A limitation of this study is reliance on qualitative data, which might have recall bias during data collection. The association made was also based on the experience of key informants. However, we minimized the bias by conducting document reviews for triangulation, peer debriefing, and member checking. Therefore, the findings from this study have been corroborated by several information resources. The data collection was conducted before the COVID-19 pandemic. Therefore, the other limitation is that this study may not represent the condition during the pandemic in which the health priority might be changed. Deploying snowball sampling to recruit participants is also a limitation of this study since the snowball may have potential recruitment bias. However, we tried to have a variation of the participants from the frontline health workers, district level government, provincial-level government, national-level government, national laboratories, and NGOs.

## Conclusion

Ensuring immunization sustainability is essential to maintain its effectiveness in the community since immunization is the most cost-effective intervention for infectious disease control. However, in a decentralized health system, the sustainability of immunization is a challenge mainly due to the competing priority, inadequate local government capacity in managing the program implementation, and vaccine hesitancy. Therefore, strong advocacy and community sensitization, and capacity building are instrumental in addressing those challenges.

## Data Availability

Data and materials supporting the findings are available from the corresponding author on reasonable request.

## Abbreviations

AEFI: Adverse Event Following Immunization
AFP: Acute Flaccid Paralysis
APBD: Anggaran Pendapatan dan Belanja Daerah/District or Province Revenue and Expenditure Budget
APBN: Anggaran Pendapatan dan Belanja Negara/National Revenue and Expenditure Budget
bOPV: Bivalent Oral Polio Vaccine
DAK: Dana Alokasi Khusus/ Specific Allocation Fund
DAU: Dana Alokasi Umum/General Allocation Fund
DGDC: Directorate General of Disease Control
DHO: District Health Office
DPHE: Directorate of Pharmacy and Health Equipment
DPT: Diphtheria, Pertussis, Tetanus
DSHQ: Directorate of Surveillance and Health Quarantine
EPI: Expanded Program in Immunization
GAVI: Global Alliance for Vaccines and Immunizations
IEC: Information, Education and Communication
IPV: Inactivated Polio Vaccine
LMICs: Low- and Middle-Income Countries
MHREC: Medical and Health Research Ethical Committee
MoH: Ministry of Health
NGO: Non-governmental Organization
NIDs: National Immunization Days
NPAFP: Non-polio Acute Flaccid Paralysis
NT: Neonatal Tetanus
PHC: Primary Health Care/Puskesmas
PHO: Provincial Health Office
SEAR: South East Asia Region
SO: Surveillance Officer
SPM: Standar Pelayanan Minimum/Minimum Service Standards
tOPV: trivalent Oral Polio Vaccine
TT: Tetanus Toxoid
VDPV: Vaccine Derived Poliovirus
VPDs: Vaccine Preventable Diseases
